# A Comparison of the **α**2/3/5 Selective Positive Allosteric Modulators L-838,417 and TPA023 in Preclinical Models of Inflammatory and Neuropathic Pain

**DOI:** 10.1155/2011/608912

**Published:** 2011-11-28

**Authors:** Sarah Nickolls, Hannah Mace, Rebecca Fish, Michelle Edye, Rachel Gurrell, Magnus Ivarsson, Tom Pitcher, Sachi Tanimoto-Mori, Denise Richardson, Catherine Sweatman, Janet Nicholson, Cameron Ward, John Jinks, Christine Bell, Kimberly Young, Huw Rees, Andrew Moss, Ross Kinloch, Gordon McMurray

**Affiliations:** Discovery Biology, Pfizer Inc., Ramsgate Road, Sandwich, Kent CT13 9NJ, UK

## Abstract

GABA_A_ receptors containing **α**2/3 subunits are current targets in the battle to develop new pain medications, as they are expressed in the spinal cord where increasing inhibitory drive should result in analgesia. However, this approach is prone to a range of side effects including sedation, cognitive impairment, and abuse as a consequence of the widespread influence of GABA. The ability to make subtype selective low-efficacy benzodiazepine compounds, which potentiate the action of GABA at specific **α** subunits, has the potential to reduce this side effect profile. In this study, we have investigated the effects of the medium-efficacy positive allosteric modulator (PAM) L-838,417 and the low-efficacy PAM TPA023 in a number of preclinical inflammatory and neuropathic pain models. We conclude that either the higher level of efficacy at **α**2/3 or efficacy at **α**5 is required for compounds to have a significant analgesic effect in a range of models, and, therefore, although the side-effect profile of compounds can be reduced compared to typical benzodiazepines, it is unlikely that it can be completely eliminated.

## 1. Introduction

GABA and glycine are the main inhibitory neurotransmitters in the CNS. GABA mediates its effects through both GABA_A_ receptors which are ligand-gated ion-channels and GABA_B_ receptors which are GPCRs. GABA_A_ receptors are heteropentameric, and the majority of those present in the CNS contain two *α*, two *β*, and a single *γ* subunit [1]. Benzodiazepines are allosteric ligands, that is, they exhibit no intrinsic activity of their own, but potentiate or inhibit the effects of GABA at receptors that contain either an *α*1, 2, 3, or 5 subunit [2]. GABA activation of GABA_A_ receptors leads to the opening of their integrated chloride channels. Chloride influx inhibits transmitter release from primary afferent terminals and hyperpolarizes spinal cord neurones, decreasing the probability of firing.

Inhibitory neurotransmission, in the spinal cord, is of great importance in pain transmission, and enhancement of inhibition leads to analgesia. Clinically, Ziconotide, an N-type calcium channel blocker which inhibits neurotransmitter release in the spinal cord, was recently approved for severe chronic pain [3]. However, its use is severely limited by CNS side effects, so there is a need for better tolerated medications. It was only recently that GABA_A_ receptors as targets for pain have gained some support from preclinical evidence, with the use of both point mutant diazepam insensitive GABA_A_ mice and subtype selective compounds. In particular, positive modulation of GABA action at *α*2 and *α*3 GABA_A_ containing receptors, in the spinal cord, results in pain relief [4]. This study looked at a combination of diazepam efficacy in point mutant mice and the efficacy of the *α*2/3/5 selective positive allosteric modulator (PAM) L-838,417 [5] in a rat chronic constriction injury (CCI) model to draw this conclusion. An additional study has added weight to the evidence, as NS11394 (which is also *α*2/3/5 selective [6]) is also analgesic in preclinical models of inflammatory and neuropathic pain [7].

Compounds, such as TPA023 [8], which has no *α*1 activity, low levels of *α*2/3 efficacy, and minimal activity at *α*5 subunits, have been shown to be anxiolytic, both preclinically and clinically [9]. However, so far all the preclinical pain studies published have used compounds with higher efficacy than TPA023. Studies using cognitive enhancing *α*5 specific NAMS suggest that PAM activity at *α*5 may be impair cognition [10]; therefore, avoidance of this activity would be an advantage in an analgesic. Furthermore, although *α*1 is likely the primary mediator of the addictive properties of benzodiazepines [11], decreasing the level of *α*2/3 activity may reduce the abuse potential of the compound [12]. We were therefore interested in whether a compound showing a lower level of *α*2/3 efficacy and minimal activity at *α*1/5, with little indication of acute clinical side effects [13] would have efficacy in preclinical pain models. For that reason, in this study, we have compared and contrasted the analgesic profile of L-838,417 (*α*1 2%, *α*2 43%, *α*3 43%, *α*5 39% compared to chlordiazepoxide [5]) and TPA023 (*α*1 0%, *α*2 11%, *α*3 21%, *α*5 5% compared to chlordiazepoxide [8]) in a wide range of preclinical neuropathic and inflammatory pain models. In addition, we have measured changes in qEEG beta frequency as a potential biomarker of *in vivo* pharmacology. We find that the lower-efficacy compound TPA023 does not exhibit a broad analgesic profile across the spectrum of preclinical pain models and that this corresponds to nonsignificant changes in the qEEG beta frequency. However, the higher-efficacy compound L-838,417 significantly reverses allodynia in the majority of the pain models tested and significantly increases qEEG beta frequency.

## 2. Materials and Methods

### 2.1. Animals

All experiments were conducted in accordance with the United Kingdom (UK) Home Office Animals (Scientific Procedures) Act (1986) and were subject to local ethical review. Experiments were performed using male Sprague Dawley rats in the light period of a twelve-hour light/dark cycle. Animals were acclimatised to the facility for at least five days prior to commencing studies, were group-housed, unless otherwise stated, and had access to food and water *ad libitum*. All surgical procedures were conducted in aseptic conditions.

### 2.2. Receptor Occupancy (RO) Studies

RO was determined using a separate cohort of animals, and these values correlated to pharmacokinetic (PK) data obtained in the studies described below. In RO studies, rats received either vehicle control, L-838,417, or TPA023 (0.3, 1, or 10 mg/kg) *p.o*. Nonspecific binding was determined in a separate group of animals by administering 5 mg/kg bretazenil *i.p.*, with a thirty-minute pretreatment time. At three mins prior to the cull, all rats were dosed *i.v*. with 10 *μ*Ci/kg [^3^H] Ro 15-1788 (flumazenil). Following euthanasia, the whole brain was removed and homogenised in 10 vol of ice-cold buffer (10 mM potassium phosphate/100 mM potassium chloride buffer, pH 7.4, 4°C) using a polytron homogeniser (setting 6 for 10 s). Three 300 *μ*L aliquots of homogenate were filtered over 0.5% v/v polyethyleneimine-(PEI-) soaked (Sigma, Poole, UK) GF/B filters (Whatman, Maidstone, Kent) to separate the bound radioactivity from the free radioactivity [14] and washed twice in 5 mL ice-cold buffer. Filters were then placed in vials, scintillation fluid added and radioactivity counted using a 3100TR TriCarb beta liquid scintillation counter (Perkin-Elmer, Cambridge, UK). Plasma samples were also collected for PK analysis. The receptor occupancy values of L-838,417 and TPA023 were determined by calculating the reduction in specific binding in drug-treated rats relative to vehicle controls. Typically, vehicle levels of radioactivity were around 2000 dpm, and non-specific (bretazenil treated) levels were around 50 dpm.

### 2.3. Behavioural Studies

All behavioural studies were conducted in a double-blind fashion. Animals were allocated to treatment groups according to their baseline scores, in order to balance groups. Plasma samples were taken in all studies for PK analysis and to extrapolate brain RO levels.

### 2.4. Complete Freund's Adjuvant-(CFA-) Induced Thermal Hyperalgesia

Following training to the testing procedure, rats (150–200 g, Charles River, UK) received an intraplantar injection of 100 *μ*g (in 100 *μ*L) CFA suspension (Sigma, Poole, UK) to the right hind paw. Behavioural studies were conducted twenty four hours later. Following acclimatisation to the testing chamber, a mobile infrared heat source (Ugo Basile, Italy) was applied directly below the plantar surface of the contralateral hind paw (for a maximum of 20 s) and paw withdrawal latency time (PWL, s) measured, using a modified method of Hargreaves et al. [15]. Three separate readings were taken and an average value calculated. This procedure was then repeated on the ipsilateral hind paw. Animals were considered to be hyperalgesic if the ipsilateral PWL value was 5 s or less. Animals were then allocated to treatment groups and received either vehicle, test compound or 100 mg/kg ibuprofen *p.o. *PWL was assessed again at one hour postdose.

### 2.5. Tibial Nerve Transection-(TNT-) Induced Static Allodynia

Tibial nerve transection was conducted using the methods previously described by Lee et al. [16]. Rats (175–200 g, Charles River, UK) were anaesthetised via an induction chamber using 2% isoflurane (Abbott, Maidenhead, UK) in oxygen. Once anaesthetised, animals were transferred to a nose cone and a homeothermic blanket system for surgery (Harvard Apparatus, Edenbridge, UK). The right common sciatic nerve was exposed via blunt dissection through the biceps femoris. The tibial nerve was tightly ligated using two ligatures placed 3 mm apart, 5 mm distal to the sciatic trifurcation. The tibial nerve was then cut and laid back in its original position. The incision was closed, and animals recovered in heated boxes before being returned to their homecages. Two weeks postsurgery, animals were habituated to test arenas and von Frey filaments (Stoelting, Wood Dale, USA) over a three-day training period. Following this training period, static allodynia was assessed using the up-down method described by Chaplan et al. [17]. In brief, von Frey filaments ranging from 0.4 to 15.0 g were applied to the plantar surface of the ipsilateral hind paw, starting with a 2.0 g filament. Filaments were then presented in an ascending or descending pattern, depending on the animal's responses, according to published methods*. *Each von Frey filament was applied until a withdrawal response was obtained, up to a maximum of six seconds. This was repeated on the contralateral hind paw. Animals were considered to be allodynic if the ipsilateral 50% paw withdrawal threshold (50% PWT) value calculated using this paradigm was 4.0 g or less. Animals were then allocated to treatment groups and received either vehicle, test compound or pregablin 20 mg/kg* p.o.* Static allodynia was assessed again at one hour postdose. Studies were conducted four to eight weeks postsurgery.

### 2.6. Chronic Constriction Injury-(CCI-) Induced Static Allodynia

Rats (175–200 g Charles River, UK) were anaesthetised and the sciatic nerve exposed as described above. CCI surgery was conducted as previously described by Bennett and Xie [18]. In brief, proximal to the sciatic trifurcation, approximately 7 mm of nerve was freed from surrounding tissue via blunt dissection and four loose ligations applied (4–0 silk), each approximately 1 mm apart. The incision was closed, and animals recovered in heated boxes before being returned to their homecages. Two weeks postsurgery, animals were habituated to test arenas and von Frey filaments as described above. Following this training period, static allodynia was assessed using the up-down method described above. Animals were considered to be allodynic if the ipsilateral 50% PWT value was 4.0 g or less. Animals were then allocated to treatment groups and received either vehicle, test compound, or pregablin 20 mg/kg* p.o.* Static allodynia was assessed again at one hour postdose. Studies were conducted at two to four weeks postsurgery.

### 2.7. Spinal Nerve Ligation-(SNL-) Induced Static Allodynia (Performed at Aptuit, Edinburgh, UK)

Rats (200–300 g, Harlan, UK) were anaesthetised using isoflurane in oxygen. The left L_6_ transverse process was removed and the L_5_ and L_6_ spinal nerves tightly ligated (6–0 silk) [19]. The incision was closed, and animals recovered before being returned to their homecages. Animals were acclimatised to the testing procedure prior to SNL surgery and retested twice after surgery as part of the training procedure. Following this training period, static allodynia was assessed two to three weeks postsurgery by applying a range of von Frey filaments from 2.0 to 26.0 g in ascending order. Each filament was applied 8–10 times at a frequency of 1 Hz. Both the contralateral and ipsilateral hind paws were assessed. Animals were considered to be allodynic if the ipsilateral paw withdrawal threshold (PWT) was 5.0 g or less. Animals were then allocated to treatment groups and received either vehicle, test compound, or pregablin 50 mg/kg *p.o.* Static allodynia was assessed again at one hour postdose.

### 2.8. Electrophysiology Studies in CCI Animals

Following CCI surgery and subsequent assessment of static allodynia, a cohort of animals were used for *in vivo* electrophysiology studies (2.5–4.5 weeks postsurgery). Animals were anaesthetised as described above. Surgery was conducted under 2.5–3.5% isoflurane. The jugular vein and carotid artery were cannulated and laminectomy performed in the lumbar enlargement region. The spinal cord was exposed, the dura removed, and the cord covered in mineral oil (Sigma, Poole, UK) at 37°C throughout the remainder of the study. Following surgery, isoflurane was decreased to 1.8–2.5% and blood pressure monitored via the carotid artery cannula. Extracellular, single-unit recordings were made using 5 MΩ fine tungsten electrodes (A-M Systems Inc., Sequim, USA) from wide dynamic range (WDR) neurones with a receptive field on the plantar surface of the ipsilateral hind paw. Neurones were characterised by intensity-dependent firing to a range of cutaneous stimuli. Action potentials were preamplified (Neurolog NL100AK headstage), amplified (Neurolog NL104A), and filtered (Neurolog NL125) (Digitimer, Welwyn Garden City, UK), and recordings digitized using a Power 1401 (CED, Cambridge, UK). Data were recorded and analysed using Spike 2 (CED, Cambridge, UK). Once identified, a noxious mechanical pinch stimulus was applied to the centre of the cell's receptive field, via an 8 cm Glover bulldog clamp (503236, WPI, Stevenage, UK). This stimulus was applied for five seconds at 10 min intervals and evoked responses recorded. The number of evoked potentials in 1–5 seconds of each stimulus application was calculated. Once stability of response was achieved, stimuli were continued in the presence of either L-838,417 or vehicle. Solutions were infused via the jugular vein at 4 mL/kg/hr over thirty mins. Blood samples were taken from the carotid artery for PK analysis. Studies were conducted in a randomised fashion.

### 2.9. Taqman Analysis of Tissue from TNT Animals

Fourteen days after surgery (and following confirmation of the development of static allodynia), a cohort of TNT-injured animals was used for analysis of KCC2 mRNA levels in the dorsal horn of the ipsilateral spinal cord. Comparative tissues were also taken from the contralateral side and from a group of sham-operated animals. Tissues were lysed using RLT buffer before extracting and purifying RNA using an RNAeasy microkit (Qiagen, Crawley, UK). The RNA quantity (A260) and purity (260/280 ratio) were assessed using spectrophotometry, and the integrity was checked using an Agilent 2100 Bioanalyser (Agilent, Winnersh, UK). Following this, a two-step amplification process was carried out, before conducting Taqman analysis.

### 2.10. Electroencephalogram (EEG) Studies

Rats (250 g, Charles River, UK) were anaesthetised using isoflurane anaesthesia as described above and implanted intraperitoneally with radio telemetric transmitters (TL11M2 F40-EET, Data Sciences International, St. Paul, Minn, USA) and with cortical EEG electrodes (stainless steel screw electrodes). These were implanted epidurally over the left parietal cortex (2.0 mm anterior and 2.0 mm lateral to lambda) and over the left frontal cortex (2.0 mm anterior and 2.0 mm lateral to bregma) for a frontal-parietal EEG recording [20]. The cortical electrodes and accompanying leads were secured to the skull by covering with dental acrylic. Animals recovered in heated boxes before being returned to their homecages (from this point animals were single-housed). EEG studies were conducted a minimum of two weeks after surgery. At the beginning of the light phase, animals received either L-838,417, TPA023, or vehicle control *p.o.* in a four-way cross-over design, so that all animals received all of the treatments, thus enabling within animal comparisons. EEG data were then immediately recorded, sampling continuously at 500 Hz for four hours with Data Sciences International hardware and Data Acquisition Gold version 3.01 software (Data Sciences International, St. Paul, Minn, USA). Data were analysed using Spike 6 (CED, Cambridge, UK). For the EEG analysis, consecutive 12-s epochs were subjected to a Fast Fourier Transform and the EEG power density within four frequency bands (*δ* 1–4 Hz; *θ* 6–9 Hz; *α* 8–13 Hz; *β* 13–40 Hz; *γ* 40–80 Hz) was calculated. Spectral analysis was performed on raw data files, which were sampled as for sleep data (512 Hz, Hanning window). Epochs containing artefacts were excluded from analysis, but otherwise, data were integrated for each frequency band, as defined above, and mean values were computed for each.

### 2.11. Quantification of the Plasma Concentrations of L-838,417 and TPA023

Quantification of L-838,417 and TPA023 in plasma was carried out using liquid chromatography-mass spectrometry (LC-MS) over a number of occasions. A typical system consisted of a binary pump (Agilent 1100 series), autoinjector (CTC PAL), and API4000 triple quadrapole mass spectrometer (Sciex). Typical HPLC conditions used a Monolith C18 column with a binary solvent system consisting of solvent mix (A) 0.027% v/v formic acid and 10 mM ammonium formate in 90 : 10 water : methanol and solvent mix (B) 0.027% v/v formic acid and 10 mM ammonium formate in 90 : 10 methanol : water. The flow rate was 1200 uL/min with the following gradient system: 0-0.1 min 0% B, increasing to 100% B at 0.45 min and holding until 2 min, returning to 0% B at 2.1 min and holding until 2.5 min. Flow was diverted to waste for the first min and after 2.4 min of each injection. The analytes were extracted from a 50 **μ**L plasma sample following the addition of 10 **μ**L of 1 **μ**g/mL internal standard solution (the two compounds were used as internal standards for each other), 300 **μ**L of pH 10 borate buffer, and 1000 **μ**L of methyl t-butyl ether (MTBE) before vortex mixing. Samples were then centrifuged at 13,000 rpm for 15 min at 4°C, before transfer of 800 **μ**L aliquots of the MTBE layer to a fresh 96-well plate which were then evaporated to dryness under N_2_ at 40°C. The samples were then reconstituted with 100 **μ**L of the mobile phase B, and up to 45 **μ**L injections were made on to the LC-MS system described. The compounds were monitored using selective reaction monitoring with Q1/Q3 transitions of 400.0/96.0 and 396.0/110.0 for L-838,417 and TPA023, respectively. The retention times of L-838,417 and TPA023 were 1.8 and 1.9 min, respectively. The concentration range of the standard curves was typically 0.5–1000 ng/mL and linear regression equations of the standard curve required correlation coefficient of >0.97 for acceptance.

### 2.12. Drugs

For RO, behavioural and EEG studies L-838,417 (L-838) and TPA023 (Pfizer, Sandwich, UK) and pregablin (Parke-Davis, Cambridge, UK) were formulated as a suspension in 0.5% methyl cellulose (Sigma, Poole, UK) vehicle. Ibuprofen (Sigma, Poole, UK) was dissolved in saline. For electrophysiology studies, L-838,417 was formulated as a solution in 18% glycerol formal (Sigma, Poole, UK), 17% solutol HS (BASF, Germany) and 65% saline vehicle. For RO studies, bretazenil was formulated as a solution in 70% polyethylene glycol (PEG) 300 (Sigma, Poole, UK), 30% saline vehicle.

### 2.13. Statistical Analysis

Data are expressed as means ± sem, unless stated. Statistical analysis of behavioural data and KCC2 mRNA levels was conducted using a One-Way Analysis of Variance test, with the exception of SNL data where a nonparametric Mann-Whitney test was used. Statistical analysis of electrophysiology data was conducted using a two-sided *t*-test. EEG data were analysed using a Restricted Maximum Likelihood (REML) analysis, followed by Fisher's post hoc analysis. In each case, treatment groups were compared to time-matched vehicle control groups.

## 3. Results

**Figure 1:**
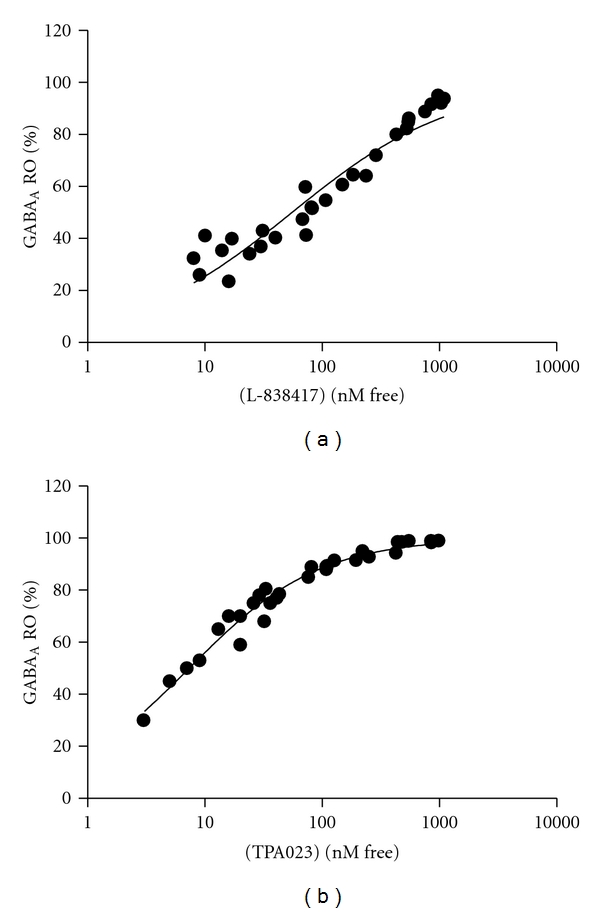
GABA_A_ receptor occupancy of (a) L-838,417 and (b) TPA023 in whole rat brain compared to drug concentrations in the plasma (nM free). Each datapoint represents an individual animal.

The aim of this study was to determine whether GABA_A_ *α*2/3 selective, positive allosteric modulators with varying efficacies *in vitro *would affect changes in *in vivo*, in preclinical pain models. In order to fully interpret and compare the data generated with the two compounds used, we first determined the brain GABA_A_ receptor occupancy of both L-838,417 and TPA023 and correlated this to nonprotein bound plasma drug levels (Figure 1). In terms of Occ50 values, TPA023 was approximately 0.3 mg/kg and L-838,417 was approximately 1 mg/kg, these data are very similar to those published by Merck [21, 22]. However, as equivalent doses did not always result in equivalent plasma exposures, in subsequent studies plasma samples were always taken for PK analysis and drug levels correlated to brain GABA_A_   receptor RO values determined from the results described above.

**Figure 2:**
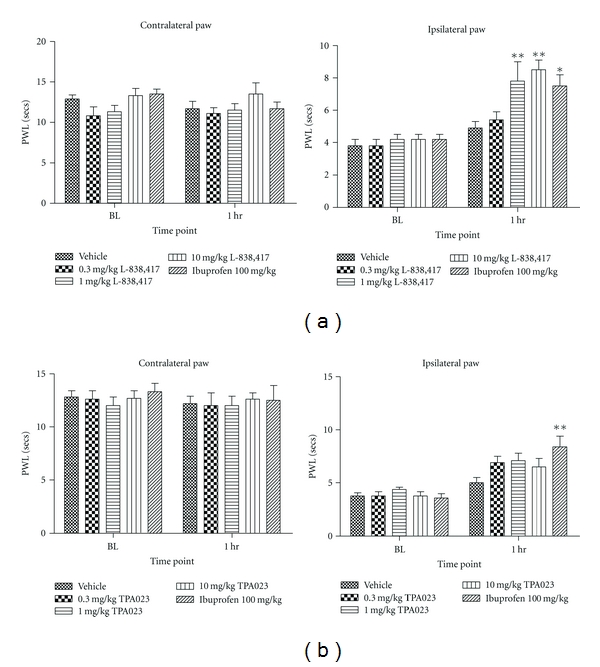
The effects of (a) L-838,417 and (b) TPA023 on complete Freund's adjuvant-induced thermal hyperalgesia in the rat. Data are means ± sem. *n* = 7-8. Data are expressed as paw withdrawal latency (PWL). BL: baseline. (∗ = *P* < 0.05, ∗∗ = *P* < 0.01, ANOVA compared to vehicle-treated group).

In terms of effects in preclinical pain models, we first examined the effects of these two modulators in a CFA-induced model of inflammatory pain. In this model, L-838,417 significantly increased PWL at 1 mg/kg (7.8 ± 1.2 s, *P* < 0.01) and 10 mg/kg (8.5 ± 0.6 s, *P* < 0.01), but was not efficacious at 0.3 mg/kg *p.o.*, when compared to vehicle control (4.9 ± 0.4 s) (Figure 2(a)). Free plasma drug levels at these doses corresponded to 64, 90 and 44% brain GABA_A_ RO, respectively. These data suggested a link between brain RO and *in vivo* efficacy in this inflammatory pain model. In the same model, no statistically significant effect was observed with TPA023 at doses up to 10 mg/kg *p.o.* (Figure 2(b)), corresponding to 98% brain RO. Therefore, it appeared that *in vitro* efficacy, in addition to *in vivo* RO, was an important factor in determining the *in vivo *efficacy of GABA_A_ modulators in pain models.

**Figure 3:**
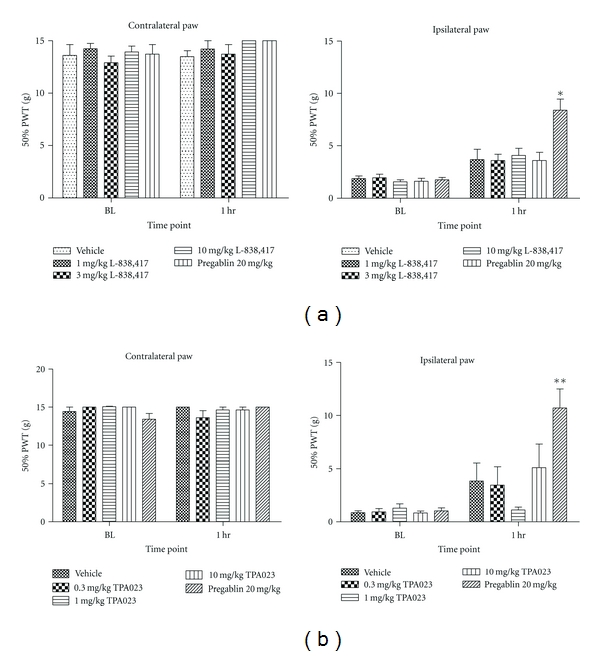
The effects of (a) L-838,417 and (b) TPA023 on tibial nerve transection-induced static allodynia in the rat. Data are means ± sem. *n* = 22. Data are expressed as 50% paw withdrawal threshold (PWT). BL: baseline. (∗∗ = *P* < 0.01, ANOVA compared to vehicle-treated group).

These compounds were then assessed in a model of neuropathic pain. Surprisingly, in contrast to the results obtained in the above inflammatory model, no significant effect was observed with either compound in the TNT neuropathic model of static allodynia (Figure 3), where the top doses correspond to 97% RO for TPA023 and 100% RO for L-838,417. This was also in contrast to data previously published by other authors in the CCI model of neuropathic pain [4].

**Figure 4:**
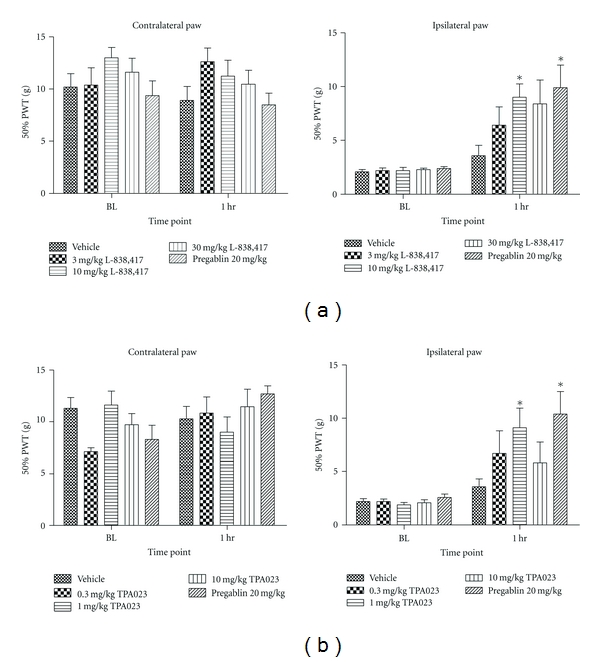
The effects of (a) L-838,417and (b) TPA023 on chronic constriction injury-induced static allodynia in the rat. Data are means ± sem. *n* = 6. Data are expressed as 50% paw withdrawal threshold (PWT). BL: baseline. (∗ = *P* < 0.05, ANOVA compared to vehicle-treated group).

In order to further investigate this discrepancy between our data and that reported by others, both compounds were then tested in a CCI model. L-838,417 significantly increased 50% PWT in this model at 10 mg/kg *p.o.* (9.0 ± 1.2 s, *P* < 0.05) compared to vehicle (3.6 ± 0.9 s) (Figure 4(a)). This dose corresponded to 94% RO. A trend for an increase in 50% PWT was also observed at 30 mg/kg* p.o*. Although this effect was not statistically significant, this value was not significantly different from that obtained at 10 mg/kg (*P* = 0.074). TPA023 also significantly increased 50% PWT in CCI animals at 1 mg/kg *p.o.* (9.1 ± 1.8 s, *P* < 0.05) with respect to vehicle control (3.6 ± 0.7 s), but this was not observed at any other dose (Figure 4(b)). This dose corresponded to 98% RO. 

**Figure 5:**
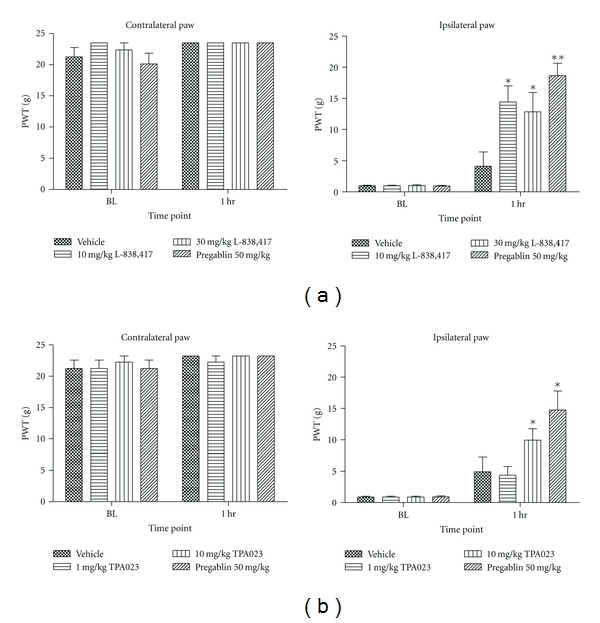
The effects of (a) L-838,417 and (b) TPA023 on spinal nerve ligation-induced static allodynia in the rat. Data are means ± sem. *n* = 10. Data are expressed as paw withdrawal threshold (PWT). BL: baseline. (∗ = *P* < 0.05, ∗∗ = *P* < 0.01 compared to vehicle-treated group. Mann-Whitney nonparametric test).

These apparent differences in the effects of GABA_A_ allosteric modulators in models of neuropathic pain were studied further by testing both compounds in an SNL model. L-838,417 significantly increased PWT in SNL animals at 10 and 30 mg/kg* p.o.* (14.4 ± 2.6 g and 12.9 ± 3.1 g, resp., *P* < 0.05 relative to vehicle control 4.1 ± 2.3 g) (Figure 5(a)). These doses corresponded to 60 and 100% RO, respectively. TPA023 was also efficacious in this model of static allodynia at 10 mg/kg *p.o.* (9.9 ± 1.8 g, *P* < 0.05) versus vehicle control (4.9 ± 2.4 g) (Figure 5(b)). This dose corresponded to 100% RO. These results suggested that when a compound with lower *in vitro* efficacy is able to exert an effect in *in vivo *pain models, it may be necessary to achieve higher brain RO levels than with a higher efficacy compound. 

**Figure 6:**
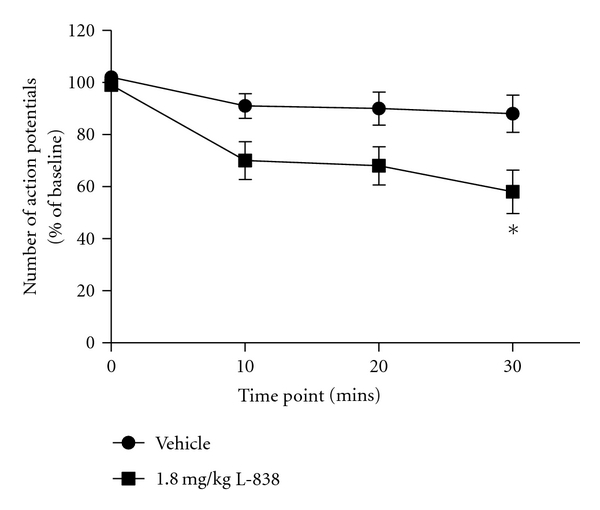
The effects of L-838,417 on pinch-evoked wide dynamic range (WDR) neurone firing in the chronic constriction injured rat. Data are means ± sem. *n* = 6–8. (∗ = *P* < 0.05 compared to vehicle-treated group, *t*-test.)

Following initial behavioural assessment, L-838,417 was also tested in CCI-injured animals in an *in vivo* electrophysiology study. The aim of this study was to determine whether GABA_A_ receptor modulation was affecting spinal nerve firing or reflex behaviours. Pinch-evoked firing of WDR neurones in CCI-injured animals was significantly decreased thirty mins after beginning *i.v.* administration of L-838,417 (58.0 ± 8.4% preinfusion number of action potentials, *P* < 0.05), relative to vehicle control (88.0 ± 7.1% preinfusion number of action potentials) (Figure 6). Drug levels at this timepoint corresponded to 86% RO. Due to the considerable number of animals utilised in these studies, we did not test TPA023 due to the weaker effect seen in the CCI behavioural study.

**Figure 7:**
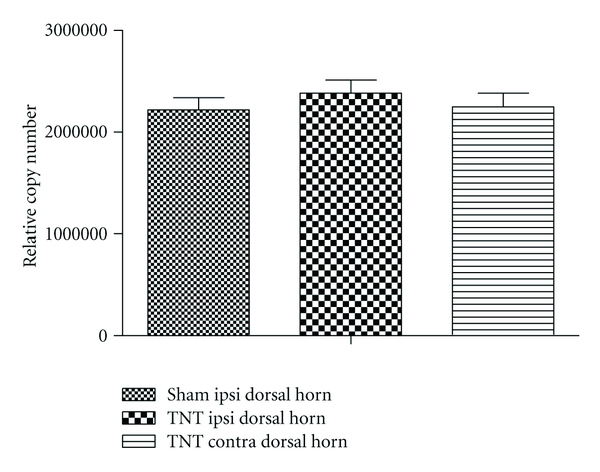
KCC2 mRNA levels in the dorsal horn of the rat spinal cord following either chronic constriction injury (CCI) or sham surgery. Data are means ± sem. *n* = 18. Ipsi: ipsilateral. Contra: contralateral.

Other groups have reported that GABA activity can be excitatory in certain conditions due to decreased levels of the potassium chloride cotransporter KCC2 [23]. In our studies, the only experimental model in which no efficacy was observed with either GABA_A_   modulator was the TNT model of neuropathic pain. We therefore examined KCC2 mRNA levels in this model using Taqman analysis. We found no significant differences in dorsal horn KCC2 levels in sham, ipsilateral TNT, and contralateral TNT tissues (Figure 7). As we observed a significant effect of GABA_A_ PAMS in the CCI and SNL model, we did not investigate any potential changes in KCC2 levels in tissues from these models.

**Figure 8:**
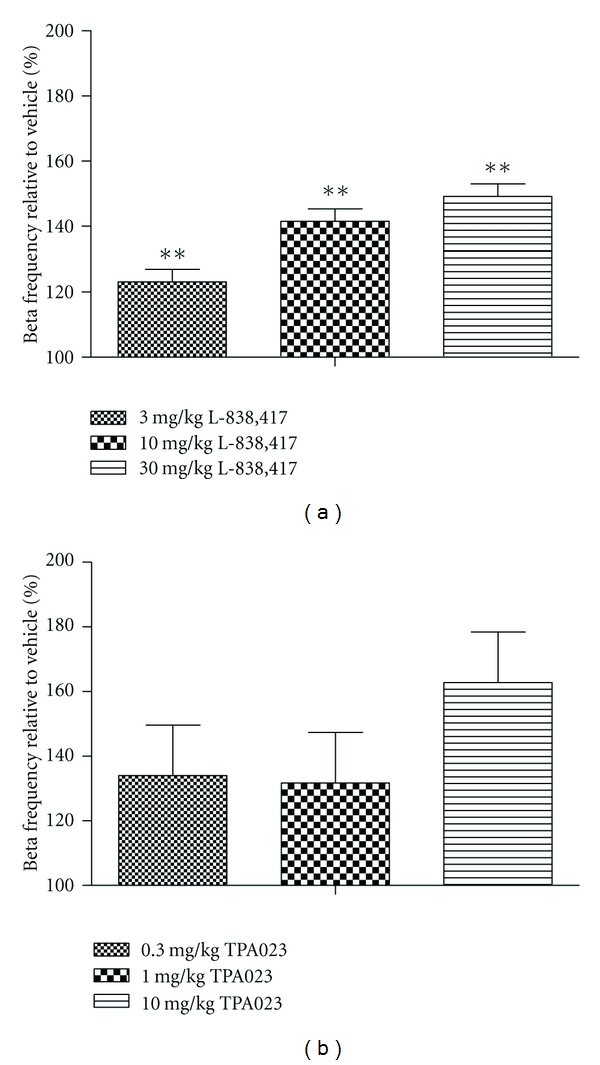
The effects of (a) L-838,417 and (b) TPA023 on quantitative electroencephalogram (EEG) in the telemetered rat. Data are expressed as the total power in the beta frequency of the EEG signal over a four-hour period, as a percentage of vehicle control. Data are means ± sed. *n* = 6. Studies were four-period crossovers, and therefore means and SED's are adjusted for period and rat effects. ∗∗ = *P* < 0.01 compared to vehicle-treated group. Restricted maximum likelihood (REML) analysis, followed by Fisher's post hoc analysis.

Finally, we studied the effects of L-838,417 and TPA023 in a quantitative EEG model, to assess the functional activation in a translatable pharmacology biomarker assay. L-838,417 dose-dependently increased the power in the beta frequency relative to vehicle control over a four-hour period (Figure 8). There appeared to be a trend towards an increase in this value with TPA023, but this was not statistically significant. PK samples could not be taken during these studies for technical reasons, so we were able to correlate these data exactly with RO. However, the three doses of each compound were targeting 50, 75 and >100% RO, respectively, and, based on experience, we are confident that we achieved 100% RO at the highest dose.

## 4. Discussion

In this paper, we have investigated the level of *α*2/3/5 efficacy required for GABA_A_ PAMS to exhibit efficacy in preclinical pain models. We have shown that L-838,417, which exhibits moderate *α*2/3/5 efficacy, exhibits a significant effect in the majority (4/5) of preclinical pain models in which it was tested. Conversely, a lower-efficacy *α*2/3 compound, TPA023, with minimal *α*5 activity, only exhibited a significant analgesic effect in two out of the four preclinical pain models investigated.

Furthermore, we have revealed that, even considering that we reached ~100% RO in all studies, the effect of the same compound across similar preclinical neuropathic pain models differs considerably. L-838,417 was able to reverse the deficit in paw withdrawal induced by either CCI or SNL, but not TNT surgery, despite behavioural testing at similar timepoints after surgery across the different models. It has been proposed that changes in chloride homeostasis, due to decreases in expression of the potassium chloride co-transporter KCC2, may cause GABA transmission to become excitatory rather than inhibitory [23, 24]. This has led to much debate, as to whether GABA_A_ PAMS will be of utility in the treatment of neuropathic pain. Due to the lack of effect of L-838,417 in our TNT model, we did investigate whether there were any changes in KCC2 expression 14 days after surgery (maximal allodynia), we found no changes in either mRNA or protein expression (data not shown) in either the ipsilateral or contralateral dorsal horn. It is possible that a deficit had normalised by this time point, as observed by G. Miletic and V. Miletic [25], but in this case, any change in chloride homeostasis should have also normalised, so we conclude that a change in KCC2 expression was not responsible for the lack of GABA_A_ PAM efficacy observed in the TNT model. Additionally, in human clinical studies, midazolam has been shown to have significant analgesic properties when given i.t in posthepatic neuralgia patients [26], suggesting that in the clinical neuropathic population, there is unlikely to be a reversal in chloride homeostasis. We do not have a conclusive explanation for the different effect of GABA_A_ PAMS across the neuropathic models, as differences between them are not well understood. The CCI model is reported to be more sensitive to mechanical stimuli than the SNL model [27], but there has not been a comprehensive study comparing which models respond best to which pharmacological approaches. It could be the case that in the TNT model, that there is a lack of inhibitory drive, and, therefore, there is no benefit in enhancing it. Often with comparing data between laboratories, it may be that slight differences in surgery, time-point after surgery tested, or even the genetic background of the animals used makes more difference to outcome, than which model is used. In our opinion, it is therefore difficult to interpret results from preclinical neuropathic models when assessing novel targets. Additionally, it is not believed that preclinical pain models accurately represent human clinical pain [28], so their value when used alone is limited.

As well as the above issues, in behavioural models, it can be difficult to separate out side effects from a true analgesic response. In particular, sedation can lead to a decrease in paw-withdrawal latency in evoked endpoints in preclinical models (internal data). Although data is always collected on the contralateral paw to try and gather globalised behavioural changes data and minimise any misinterpretation of the ipislateral data, because of the difference in baseline between the two paws, it is possible that there may still be some side effect interference. Indeed, reviewing the published data on L-838,417, we consider that, although no statistics are given, it appears that there is a deficit in the dark phase of the fear-potentiated startle test [5]. Furthermore, we have also observed that zolpidem-trained rats generalise slightly when given L-838,417 (data not shown). Both of these observations suggest that L-838,417 may not completely lack *in vivo* activity at the *α1* subunit. In addition, although rotorod performance is less impaired by diazepam in the *α*1 KI mice, there is still a deficit in performance at high doses [5], pointing towards a motor-impairment effect, possibly muscle relaxation, mediated through one of the other *α* subunits. Additionally, TPA023B which has a similar *in vitro* profile to L-838,417 reportedly causes effects such as flaccid body tone in conscious animals [29]. We consider, however, that the data obtained in the CCI-wide dynamic range (WDR) study provide unequivocal evidence that GABA_A_ PAMS do significantly affect pain signalling. This is because recordings are from individual spinal WDR neurones, which respond to a range of sensory stimuli. Increasing intensities of stimulation cause increasing cell firing, with maximal firing caused by noxious stimuli. These cells signal pain (intensity and location) to the brain. The CCI-WDR assay is designed to measure the effects of compounds on activity evoked in WDR neurones by peripheral stimuli that are in the noxious range, and L-838,417 was able to reduce the amount of nerve firing caused by a pinch stimulus in these animals.

With regards to receptor occupancy (RO), even the higher activity compound L-838,417 required at least 60% RO in preclinical models to exhibit significant efficacy. Indeed, the SNL model appeared to be the most sensitive of the neuropathic models with regards detecting an analgesic effect, with L-838,417 requiring 60% RO and TPA023 requiring 100%, which correlates well with their different *in vitro* profiles. Conversely, in the CFA-thermal inflammatory model although L-838,417 again had an analgesic effect at 60% RO, TPA023 was ineffective in this model. How receptor occupancy requirements will translate from the preclinical to clinical setting is difficult to estimate. The optimistic viewpoint would be that similar to the sedative and anxiolytic properties of classical benzodiazepines, such as zolpidem and lorazepam, that lower occupancy is required in the clinical setting to achieve significant effects [30, 31], but this hypothesis needs clinical data to support it. In phase 1 safety studies, TPA023 was shown to be well tolerated up to approximately 60% receptor occupancy [9], in addition TPA023B which has a similar *in vitro* profile to L-838,417 was also well tolerated up to approximately 60% receptor occupancy [32]. This was an acute study, however, and, at higher doses, the reported side effect profile of TPA023B appears to be worse than that of TPA023, with clear sedative and ataxic effects.

Overall, our data cannot differentiate whether it is the higher *α*2/3 activity exhibited by L-838,417 compared to TPA023 that leads to a more robust analgesic profile in preclinical pain models, or whether the difference is due to the *α*5 activity present in L-838,417. Both of the functionally selective GABA_A_ compounds currently published in the literature which exhibit a significant effect in preclinical pain models do show reasonable activity at *α*5 [4, 7]. In addition, in knockin mice, the efficacy of diazepam against mechanical and heat allodynia was significantly reduced in the *α*5 KI's after CCI surgery [4], suggesting that this subunit does play some role in mediating the analgesic effects of these compounds. Hopefully, advances in medicinal chemistry and the production of more selective compounds will allow this question to be answered in the near future.

Finally, in order to have a translatable measure of *α*2 activity *in vivo*, we also investigated the changes in qEEG beta frequency produced by L-838,417 and TPA023. Preclinically, the beta frequency is considered to be a marker of *α*2 activity, as qEEG studies have shown that the change in diazepam-induced beta frequency remains unaffected in *α*1 or *α*3 knockin mice [33–35], but was reduced in mice with diazpam-insensitive *α*2 subunits. In these mice, *α*2 subunits were functional (i.e., they respond normally to GABA) but are diazepam insensitive. Clinical qEEG changes have been reported for a number of benzodiazepine compounds [36–38], although the clinical data supporting a correlation between beta frequency and *α*2 has not, at this time, been generated. Our preclinical qEEG data correlate well with the reported *in vitro* activity of the two compounds and to some extent mimic the effects observed in our preclinical pain models, with L-838,417 exhibiting a significant change in beta frequency in a dose-related fashion, but TPA023 exhibiting a nonsignificant trend to increase. We propose that changes in qEEG beta frequency may be an appropriate pharmacological biomarker for *α*2 selective GABA_A_ PAMS.

## 5. Conclusions

We conclude that GABA_A_ functionally selective PAMSs are likely to have broad utility in treating clinical pain. We consider, however, it is unlikely that a low-efficacy compound such as TPA023 will show sufficient efficacy in the clinic. The balance of an increasing side effect profile and efficacy will therefore have to be carefully considered when taking compounds into clinical testing. We also recommend the use of qEEG as an early marker of pharmacology in the clinical setting.
